# Decoding the complex receptor landscape of enterovirus D68

**DOI:** 10.1371/journal.ppat.1014231

**Published:** 2026-05-26

**Authors:** Mengyang Zhao, Leiliang Zhang

**Affiliations:** 1 Department of Clinical Laboratory Medicine, The First Affiliated Hospital of Shandong First Medical University & Shandong Provincial Qianfoshan Hospital, Jinan, Shandong, China; 2 Department of Pathogen Biology, School of Clinical and Basic Medical Sciences, Shandong First Medical University & Shandong Academy of Medical Sciences, Jinan, Shandong, China; Washington University School of Medicine in Saint Louis: Washington University in St Louis School of Medicine, UNITED STATES OF AMERICA

## Abstract

Enterovirus D68 (EV-D68) is an emerging pathogen associated with severe respiratory diseases and central nervous system complications. This pearl summarizes the roles of glycan receptors, such as sialic acid, and protein receptors like ICAM-5 and MFSD6 in EV-D68 infection. MFSD6 serves as a primary receptor due to its widespread distribution in tissues, while sialic acid and ICAM-5 play a synergistic role in facilitating invasion across various tissues. We have elaborated on the correlation between receptor distribution, tissue tropism, and pathogenicity. The complex receptor system of EV-D68 enhances its pathogenicity by facilitating multi-tissue invasion and allowing the virus to adapt to various microenvironments. This adaptability provides insights into distinct receptor preferences and their associations with pathogenic outcomes, where specific receptor binding can influence the severity of infection. Additionally, we discuss the effectiveness of receptor-targeted inhibitors and the potential for therapeutic interventions aimed at disrupting receptor-virus interactions. Our pearl underscores the multifaceted nature of EV-D68 receptors and their implications for the development of novel antiviral strategies.

## Q: What are the known receptors of EV-D68?

**Fig 1:**
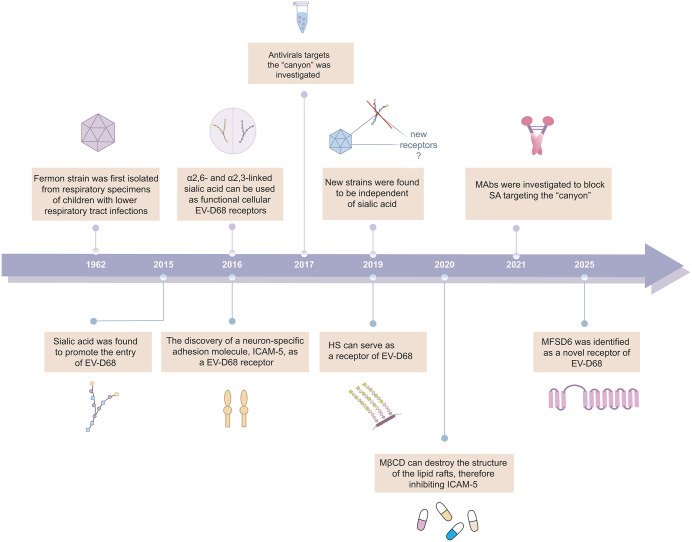
Timeline of milestones in the research on EV-D68 receptor.

**A:** Enterovirus D68 (EV-D68) is an emerging enterovirus responsible for severe respiratory diseases and central nervous system (CNS) complications, such as acute flaccid myelitis (AFM) [1]. Belonging to the the genus *Enterovirus* in the family *Picornaviridae*, EV-D68 is a non-enveloped virus that carries a positive-sense single-stranded RNA [2]. This virus has an icosahedral structure composed of 60 copies of four capsid (VP1 to VP4), with VP1 as the basis for serotype classification, and VP1, VP2 and VP3 mediating interactions with various viral receptors [2]. The association of this virus with severe respiratory and CNS diseases distinguishes it from other enteroviruses, highlighting its uniqueness. Currently, the identified entry factors for EV-D68 include glycan receptors, such as sialic acid (SA) and sulfated glycosaminoglycans (sGAGs), as well as protein receptors like intracellular adhesion molecule-5 (ICAM-5) and major facilitator superfamily domain-containing 6 (MFSD6) (Fig 1) [3–8].

Research also reveals distinct receptor-binding phenotypes among EV-D68 strains. For instance, the A2/2018 strain exhibits a strong preference for ganglioside structures, particularly disialylated gangliosides (GD3, GD1c, GT1a, and GQ1b), which carry α2,8-linked SA at the penultimate residue. This indicates that glycolipids can function as entry receptors [7]. Inhibiting glycolipid biosynthesis significantly reduces the infectivity of the A2/2018 strain, while the removal of SA diminishes the infectivity of all strains to levels comparable to those caused by replication inhibitors [7]. This underscores the critical role of SA and the necessity of glycolipids for the entry of EV-D68.

## Q: How do glycan and protein receptors facilitate the multistep process of EV-D68 viral entry?

**Fig 2:**
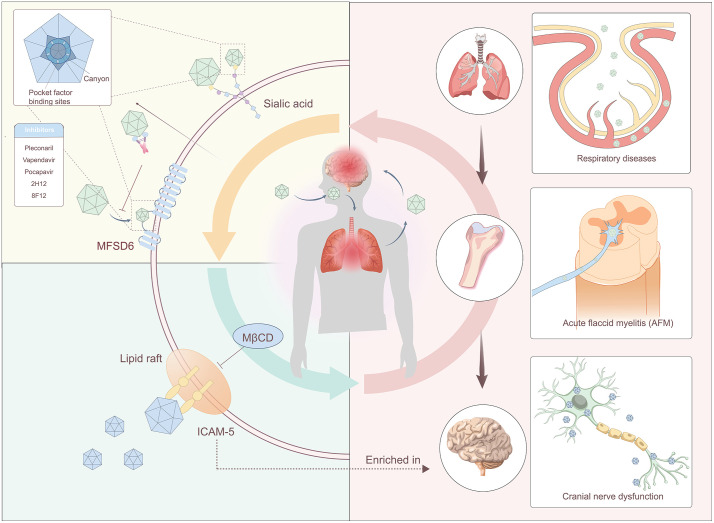
Receptor-mediated infection processes and targeted inhibitors of EV-D68. The initial viral attachment is mediated by host sialic acid (SA), which engages the viral canyon region and binding sites for pocket factors, facilitating host cell association. Small-molecule inhibitors and monoclonal antibodies specifically target the canyon, disrupting the virus-SA interaction to block attachment. EV-D68 exhibits tissue-specific infection via distinct receptors. In the CNS, the virus traffics to the spinal cord through nerve fibers, leading to acute flaccid myelitis (AFM) by engaging the neuron-enriched receptor ICAM-5, which is localized within lipid rafts. Methyl-β-cyclodextrin (MβCD) disrupts lipid raft architecture, thereby inhibiting ICAM-5-dependent viral entry. In the respiratory tract, EV-D68 utilizes the host receptor MFSD6, while MFSD6-Fc(CH₃) has been designed to antagonize this infection pathway.

**A:** EV-D68 has a complex receptor system that serves distinct functions. SA mediates EV-D68 attachment by binding to the canyon on the virus surface, which influences the pocket factor and induces a cascade of conformational changes in the virus. This interaction establishes a prerequisite for viral entry, particularly in historical prototype strains such as Fermon (Fig 2) [6]. However, the discovery of some SA-independent strains suggests that MFSD6 may present a new infection route [3,4].

ICAM-5 functions as a receptor mediating adhesion and entry of EV-D68. The critical role of the Asn54 site (a glycosylation site) of ICAM-5 in EV-D68 infection has been demonstrated by reduced viral titer and cytopathic effect after mutation [5]. Studies in cultured human cell lines show enhanced viral replication in the presence of ICAM-5 for both SA-dependent and -independent EV-D68 strains [5]. Importantly, for the Fermon strain, ICAM-5 can exert its function only in the presence of SA, indicating that SA plays a crucial role in ICAM-5-mediated EV-D68 Fermon infection. This potential synergistic interaction is also evident [5]. However, the specific tropism of ICAM-5 limits its therapeutic value in respiratory diseases and AFM [5].

MFSD6 is a novel receptor that facilitates both viral attachment and internalization, targeting various tissues, including the respiratory tract (e.g., nasal epithelium and lungs), the central nervous system, and the gastrointestinal tract. It may play a significant role in subsequent pathogenesis [3,4]. Its L3 domain acts as the main binding site, with multiple glycosylation sites functioning collaboratively. MFSD6 is localized not only on the plasma membrane but also in late endosomes and lysosomes, indicating its significant role in infection and endocytosis processes [3,4]. Notably, inhibiting MFSD6 results in a 90% decrease in viral attachment, suggesting it may serve as a principal receptor within a complex receptor network involved in infection [3,4].

## Q: What is the relationship between receptor preference and pathogenicity?

**A:** The clinical symptoms associated with EV-D68 primarily include respiratory and neurological disorders, with respiratory manifestations predominating [2]. In mild or subclinical EV-D68 infections, the virus predominantly targets the upper respiratory tract (URT), leading to symptoms such as fever and cough [9,10]. Conversely, if the virus spreads to the lower respiratory tract (LRT), it can result in more severe conditions, including dyspnea, wheezing, pneumonia, and hypoxia [9,10]. In severe cases, this may necessitate ventilator support and intensive care. A prior history of asthma or recurrent wheezing is a key risk factor for exacerbations of asthma or pneumonia caused by EV-D68 infection [9]. Its pathogenic characteristics are closely linked to the distribution of receptors, including MFSD6, SA, and ICAM-5 [3,5,6].

Both α2,6-linked SA and α2,3-linked SA serve as essential functional receptors for EV-D68 [11]. Notably, EV-D68 exhibits a stronger affinity for α2,6-linked SA (NeuAcα2,6Gal) than for α2,3-linked SA (NeuAcα2,3Gal). Since α2,6-linked SA is the primary type found in the human URT, EV-D68 may preferentially infect the URT over the LRT [1,12]. Besides SA, MFSD6 is also widely expressed in many tissues, including respiratory epithelial cells [3,4]. During viral attachment, SA and MFSD6 target distinct regions of the canyon and synergistically mediate viral entry without interfering with each other [3,6]. In addition to attachment, MFSD6 plays a critical role in post-entry steps such as internalization, suggesting that it may significantly contribute to respiratory pathogenesis [3].

Some infected individuals may experience acute flaccid paralysis and/or cranial nerve dysfunction, which are neurological manifestations of the infection. ICAM-5, located in the telencephalon, is likely associated with encephalitis and cranial nerve damage [5,10]. Despite its importance, its distribution limits its ability to motor neurons, and its neuropathogenic mechanisms remain to be fully elucidated [7]. It is noteworthy that the knockout of the MFSD6 gene reduced EV-D68 infection in neural cells differentiated from primary normal human induced pluripotent stem cells (iPSCs) [3]. Furthermore, the expression levels of MFSD6 significantly fluctuate in response to changes in the energy intake status of mice, indicating its involvement in the regulation of energy homeostasis within the CNS. This expression is dynamically regulated by the energy metabolism microenvironment of neuronal cells [13], suggesting that MFSD6 may play a primary role in the neurovirulence of EV-D68.

SA and MFSD6 serve as the initial attachment receptors, facilitating the virus’s invasion of respiratory epithelial cells, with MFSD6 further anchoring the virus and mediating its entry into these cells [3,4,7]. Unlike other enteroviruses, EV-D68 is transmitted through inhalation of aerosol particles or manual transfer of infectious material from environmental surfaces to the respiratory tract, rather than through fecal-oral transmission (Fig 2) [10]. This synergistic relationship ensures efficient invasion of the respiratory tract, allowing for initial infection and viral replication [3,4,7]. Given the widespread distribution of MFSD6 and the presence of ICAM-5 in the telencephalon, EV-D68 first establishes infection in the lungs after invading the nasopharynx, subsequently spreading to peripheral tissues via the bloodstream, particularly the muscles [14,15]. The virus replicates within muscle fibers and is released, potentially crossing the neuromuscular junction to enter motor neuron axon terminals through receptor-mediated endocytosis. It then travels retrogradely within endosomes via dynein-mediated fast axonal transport to reach the somata of spinal motor neurons [2]. This retrograde transmission of EV-D68 across the neuromuscular junction, followed by retrograde axonal transport, represents the indirect mechanism by which the virus induces AFM and myelitis (Fig 2). When the virus breaches the blood-brain barrier, it may induce cerebral symptoms potentially associated with MFSD6 and ICAM-5 [3,5].

Receptor usage directly influences viral tissue tropism and infection efficiency, which subsequently affects viral load in target tissues. Contemporary circulating EV-D68 strains that utilize MFSD6 as their primary functional receptor can effectively infect both respiratory epithelial cells and spinal motor neurons, resulting in significantly elevated viral loads in the respiratory tract and central nervous system (CNS) [16]. In contrast, the prototype strain Fermon relies on SA, exhibiting limited infection efficiency and low viral loads. Furthermore, viral load closely correlates with disease severity. High viral loads in the spinal cord cause severe damage to motor neurons, leading to acute flaccid myelitis (AFM) and increased mortality rates. Conversely, the administration of neutralizing antibodies that block receptor binding significantly reduces viral loads and markedly alleviates clinical symptoms and mortality, thereby supporting a causal link among receptor usage, viral load, and disease severity. In terms of transmissibility, effective receptor usage enhances viral replication in the respiratory tract, resulting in increased viral shedding, which promotes person-to-person transmission. The receptor adaptation of contemporary strains improves their fitness within the human airway, contributing to widespread outbreaks and periodic circulation.

## Q: What is the significance of EV-D68’s multi-receptor system?

**A:** EV-D68 possesses a unique multi-receptor system, which is highly significant for the virus. The multi-receptor system expands the range of target cells for viral infection, allowing EV-D68 to achieve a “multi-tissue invasion” strategy and circumvent the limitations of a single receptor’s expression (Fig 2). Additionally, this system enhances the virus’s environmental adaptability. Children are the primary susceptible population for EV-D68 [9], and the virus can efficiently infect them through both SA and MFSD6 [3,4,6]. Conversely, the respiratory mucosa in adults is more mature, potentially leading to decreased expression of SA-related receptors [17]. However, MFSD6 remains stably expressed, allowing the virus to maintain infectivity, even if symptoms are generally mild [18].

The multi-receptor system prevents the virus from being eliminated based on the expression differences of a single receptor among different age groups [19]. Additionally, the microenvironments of respiratory and neural tissues vary significantly in terms of pH levels, ion concentrations, and protein modification profiles [6]. A single receptor may lose binding activity due to these environmental factors. For instance, SA is vulnerable to degradation in acidic environments, whereas MFSD6, as a protein receptor, exhibits greater tolerance to environmental changes [3,4,6]. ICAM-5, on the other hand, is highly expressed only in specific neural microenvironments [5]. This multi-receptor system allows the virus to find a compatible receptor across various microenvironments, ensuring that the infection process is not inhibited by external factors.

In contrast to a single-receptor system where binding sites are highly concentrated, making the virus easier for the immune system to recognize and produce targeted antibodies, a multi-receptor system distributes the virus’s binding sites across different receptors. This distribution complicates the immune system’s ability to generate effective neutralizing antibodies against all receptors simultaneously [19]. Even when novel viral strains emerge that are SA-independent, the virus can still achieve stable infection through MFSD6, reducing the likelihood of being “completely blocked” and extending its survival and replication time within the host [3,4].

The long-evolved multi-receptor system of EV-D68 maximizes its infection and pathogenicity capabilities. This adaptability explains why EV-D68 can persistently circulate globally and cause a range of diseases, from common respiratory symptoms to severe neuropathies. The multi-receptor system is essential for the virus’s daptability to hosts, maintenance of transmissibility, and retention of pathogenicity. EV-D68 has evolved a broad and flexible repertoire of receptors, potentially driven by immune pressure, host adaptation. As population immunity against SA-utilizing strains increases, EV-D68 encounters selective pressure that favors enhanced dependence on SA-independent pathways and the use of recently identified receptors such as MFSD6 in the respiratory and nervous systems [4,20]. It is speculated that such adaptive changes allow the virus to evade pre-existing immunity, thus supporting its continued circulation. By employing both SA and MFSD6, EV-D68 can infect with high efficiency the respiratory tract and central nervous system. This dual receptor usage supports robust replication in both tissues, presumably enhancing the virus’s overall fitness and adaptability. Furthermore, the broad range of receptor usage may facilitate sustained transmission and persistence within the human population. The diversity in receptor utilization could increase viral stability in the airways, potentially improve transmissibility, and might enable seasonal outbreaks, collectively indicating a mechanism underlying the long-term persistence of EV-D68 in humans.

## Q: What is the efficiency of receptor-targeted inhibitors?

**A:** Currently, several receptor-targeting SA can bind to the canyon region of the virus, termed capsid binders. These compounds replace the pocket factors within the canyon, thereby preventing the virus from uncoating, a crucial step in which the virus releases its genetic material into the host cell to initiate replication [21] (Fig 2). DAS181, an enzymatic compound that cleaves sialidases, effectively targets both α2,6-linked and α2,3-linked SA, inhibiting viral replication *in vitro*, though its efficacy *in vivo* remains unverified [21,22]. Clinical trials for pleconaril, vapendavir (also known as BTA798), and pocapavir (also known as V-073) are currently underway or have recently concluded, demonstrating efficacy in reducing mortality rates among neonates [21,22] (Fig 2). However, the rapid emergence of drug resistance poses a critical challenge to further development, making the search for new therapeutic agents urgent. Furthermore, pleconaril has been associated with various adverse effects [21]. Analogues of pleconaril have been developed but primarily target respiratory manifestations of infection, with limited effectiveness against neurological symptoms [21,22].

Two monoclonal antibodies, 2H12 and 8F12, generated using EV-D68 virus-like particles (VLPs), exhibit a balanced and potent neutralizing effect (Fig 2). Both antibodies bind to the canyon region on the viral capsid, inducing steric hindrance that interferes with EV-D68 binding to attachment receptors [23]. To date, four neutralizing antigenic sites have been identified in close proximity to the canyon, displaying varying degrees of sequence variation. Identifying these four antigenic sites on the EV-D68 capsid enhances our understanding of how neutralizing antibodies recognize EV-D68 and uncovers the molecular mechanisms underpinning viral evolution and immune evasion [24].

EV-D68 promotes the translocation of its receptor, ICAM-5, to the lipid rafts of infected cell membranes. Targeting ICAM-5, Methyl-β-cyclodextrin (MβCD) disrupts lipid rafts [25] (Fig 2). After MβCD treatment, the colocalization of viral particles and ICAM-5 is completely abrogated, inhibiting EV-D68 replication by disrupting the aggregation of viral particles and ICAM-5 within lipid rafts [26].

Fusion proteins such as MFSD6-Fc and Fc-MFSD6 (L3) have been developed, incorporating key extracellular domains or the third extracellular loop (L3) of MFSD6, acting as decoy receptors [3,4,18] (Fig 2). These constructs specifically bind to EV-D68 virions and effectively block interactions between the virus and endogenous MFSD6 on host cell surfaces, preventing infection of respiratory epithelial cells by both SA-dependent and -independent EV-D68 strains and inhibiting viral invasion of neurorelated cells, demonstrating notable antiviral efficacy in both cellular and animal models [3,4,18]. Moreover, researchers have identified the core amino acid residues mediating the virus-MFSD6 interaction utilized cryo-electron microscopy, providing a structural basis for optimizing this neutralization strategy [3]. Collectively, these findings support developing MFSD6-targeted interventions, including antibody therapies and decoy receptor-based agents, and highlight MFSD6’s pivotal role in antiviral research.

## Q: What are the main conclusions and future research directions regarding EV-D68 receptor interactions?

**A:** In recent years, research on the receptors of EV-D68 has been continuously emerging. In this pearl, we summarize the multi-receptor system of EV-D68, discuss the roles of the major receptors MFSD6, SA, and ICAM-5 in viral entry, and analyze their associations with viral pathogenicity. Nevertheless, numerous questions remain to be elucidated.

The ICAM family has long been considered a crucial factor in nervous system infections. However, its restricted distribution prevents it from fully accounting for the pathogenesis of AFM. In the present study, we describe the infection pattern of EV-D68. The virus initiates infection via the respiratory tract and enters spinal motor neurons through retrograde axonal transport. ICAM-5 exhibits only faint punctate expression in the soma and dendrites but is absent in axons [16]. Since EV-D68 infection begins in the axons, ICAM-5 cannot participate in axonal infection and retrograde transport. Furthermore, ICAM-5 protein is not expressed in the cervical spinal cord of human children, with high expression restricted to the cerebral cortex [16]. This suggests the existence of an unidentified neuronal receptor with widespread distribution, likely MFSD6. Based on existing receptor research, MFSD6 may serve as a main receptor responsible for neuropathogenesis, mediating viral entry and subsequent endocytosis. Within the central nervous system, and especially in the telencephalon, ICAM-5 may also function as a receptor, potentially contributing to severe clinical manifestations such as cranial nerve injury and encephalitis. However, as previously mentioned, further experimental evidence is required to clarify whether receptor switching occurs.

We propose that EV-D68 may exhibit receptor usage plasticity, which is closely associated with its tissue tropism and environmental adaptability. For instance, in the respiratory system, SA mediates the virus’s initial attachment, while MFSD6 is primarily responsible for subsequent efficient viral entry into cells. Although MFSD6 contributes to viral attachment, it does not induce capsid conformational changes at the early attachment stage, but facilitates RNA release and uncoating at later stages or under endosomal acidic conditions [3,4]. In the spinal cord of the central nervous system, MFSD6 serves as the core receptor that dominates viral infection of motor neurons, while SA and ICAM-5 can act as backup receptors to exert auxiliary effects in regions where they are still expressed. This plasticity in receptor switching enables the virus to adapt more effectively to variations in receptor expression among different cell subsets within the same tissue, thereby expanding its infection range and enhancing its pathogenic potential. Future studies could utilize single-cell sequencing combined with viral tracing techniques to better verify the patterns of viral receptor switching among different cell populations within a single tissue.

Additionally, while some *in vitro* cell line studies have reported that EV-D68 can achieve infection through heparan sulfate (HS) binding, this phenomenon is only observed in immortalized cell lines such as HeLa and RD. It is likely an artifact generated during cell culture and does not represent the natural infection mode of the virus *in vivo*. More importantly, no HS binding-associated infectivity has been detected in studies of clinically isolated circulating strains, confirming that this binding mode is irrelevant to the actual infection characteristics of clinical circulating strains.
